# Pharmacologically upregulated carcinoembryonic antigen-expression enhances the cytolytic activity of genetically-modified chimeric antigen receptor NK-92MI against colorectal cancer cells

**DOI:** 10.1186/s12865-018-0262-z

**Published:** 2018-08-03

**Authors:** Masayuki Shiozawa, Chuan-Hsin Chang, Yi-Chun Huang, Yi-Ching Chen, Mau-Shin Chi, Hsu-Chao Hao, Yue-Cune Chang, Satoru Takeda, Kwan-Hwa Chi, Yu-Shan Wang

**Affiliations:** 1 Department of Obstetrics and Gynecology, Juntendo University Hospital, 3-1-3 Hongo, Bunkyo-ku, Tokyo, Japan; 2 Department of Radiation Therapy and Oncology, Shin Kong Wu Ho-Su Memorial Hospital, No.95, Wenchang Road, Shilin District, Taipei, Taiwan; 3 Department of Research and Development, Johnpro Biotech Inc., 2F., No.118, Hougang St., Shilin Dist., Taipei City, Taiwan; 4 Institute of Molecular Medicine and Bioengineering, National Chiao Tung University, Room 117 Lab Building 1, 75 Bo-Ai Street, Hsinchu, Taiwan; 5 Department of Biotechnology, Hungkuang University, No. 1018, Sec. 6, Taiwan Boulevard, Shalu District, Taichung City, Taiwan; 6 Department of Mathematics, Tamkang University, No.151, Yingzhuan Rd., Tamsui Dist., New Taipei City, Taiwan; 7 Institute of Veterinary Clinical Science, School of Veterinary Medicine, National Taiwan University, Taipei, Taiwan; 8 Department of Biomedical Imaging and Radiological Sciences, National Yang-Ming University, Taipei, Taiwan

**Keywords:** Natural killer cell, NK-92MI, Chimeric antigen receptor (CAR), Carcinoembryonic antigen (CEA), Cellular immunotherapy

## Abstract

**Background:**

The natural killer cell line, NK-92MI, is cytotoxic against various types of cancer. The aim of this study was to develop chimeric antigen receptor-modified (CAR) NK-92MI cells targeting carcinoembryonic antigen-expressing (CEA) tumours and increase killing efficacy by pharmacologically modifying CEA-expression.

**Result:**

We generated anti-CEA-CAR NK-92MI cells by retroviral vector transduction. This genetically-modified cell line recognised and lysed high CEA-expressing tumour cell lines (LS174T) at 47.54 ± 12.60% and moderate CEA-expressing tumour cell lines (WiDr) at 31.14 ± 16.92% at a 5:1 effector: target (E/T) ratio. The cell line did not lyse low CEA-expressing tumour cells (HCT116) as they did their parental cells (NK-92MI cells). The histone deacetylase-inhibitor (HDAC) sodium butyrate (NaB) and the methylation-inhibitor 5-azacytidine (5-AZA), as epigenetic modifiers, induced CEA-expression in HCT116 and WiDr cells. Although the IC_50_ of 5 fluorouracil (5-FU) increased, both cell lines showed collateral sensitivity to anti-CEA-CAR NK-92MI cells. The cytolytic function of anti-CEA-CAR NK-92MI cells was increased from 22.99 ± 2.04% of lysis background to 69.20 ± 11.92% after NaB treatment, and 69.70 ± 9.93% after 5-AZA treatment, at a 10:1 E/T ratio in HCT116 cells. The WiDr cells showed similar trend, from 22.99 ± 4.01% of lysis background to 70.69 ± 10.19% after NaB treatment, and 59.44 ± 10.92% after 5-AZA treatment, at a 10:1 E/T ratio.

**Conclusions:**

This data indicates that the effector-ability of anti-CEA-CAR NK-92MI increased in a CEA-dependent manner. The combination of epigenetic-modifiers like HDAC-inhibitors, methylation-inhibitors, and adoptive-transfer of ex vivo-expanded allogeneic-NK cells may be clinically applicable to patients with in 5-FU resistant condition.

## Background

Human natural killer cells (NK) play an important role in innate immune defence against viral infections and malignant cells [1, 2]. NK cells do not require antigen representation for target recognition. They show tumour cytotoxicity in a major histocompatibility complex-unrestricted (MHC-unrestricted) manner [1, 2]. Cancers induce NK cell dysfunction, resulting in reduced proliferation of NK cells, decreased infiltration of tumours, and/or defective cytokine production. Cancer cells also evade NK cell attack via lowering expressions of activating receptors and intracellular signalling molecules, and/or overexpression of inhibitory receptors [3].

The adoptive transfer of NK cells has been used widely in clinical trials [4]. Sources of NK cells used in adoptive transfer include primary autologous (patients) [5], allogeneic (healthy donor) [6], umbilical cord blood [7], induced pluripotent stem cells (iPSC) [8], and NK cell lines [9]. NK-92 cells have undergone extensive preclinical development [10] and have completed phase I trials in cancer patients [9]. Unlike primary NK cells, NK-92 cells express almost no inhibitory killer cell immunoglobulin-like receptors (KIRs) [11]. NK-92MI is an interleukin-2-independent (IL-2-independent) cell line derived from NK-92 by transfection of human IL-2 cDNA. It has the same characteristics as activated-NK cells and its parental NK-92 cells [12]. It has been shown that both NK-92 and NK-92MI cells are highly cytotoxic to human melanoma cells both in vitro and in vivo [13, 14]. Genetically-modified effector cells with a chimeric antigen receptor (CAR) were chosen with the intention of enhancing their reactivity against antigen-expressing tumour cells [15–17]. However, chimeric antigen receptor T-cell (CAR-T) treatments are associated with adverse events, mostly commonly eliciting Cytokine Release Syndrome (CRS), a systemic inflammatory response that can lead to widespread organ dysfunction and death [18, 19]. NK cell lines may be alternative cytotoxic effectors for CAR-driven tumour cell-specific cytolysis [20, 21]. The limited life span of NK cells may make them safer than T cells [15]. In recent years several CAR-modified NK-92 and NK-92MI cells have been developed. They were highly cytotoxic both in vitro and in vivo [22–27]. However, developments in anti-CEA-CAR-modified NK-92 and NK-92MI cells have not been well-characterized. CEA is expressed in various cancers and has been used in CEA-CAR-T therapy [28]. CEA-overexpression may contribute to human cancer progression by inhibiting cell-differentiation, apoptosis, and anoikis [29, 30]. CEA-expression level per cell may be a suitable biomarker for predicting tumour response to chemotherapy in colorectal cancer [32]. We proposed that CEA-targeted adoptive immunotherapy is a good example of collateral sensitivity to 5-FU-resistant CEA-expression in tumours.

In this study, we successfully transduced NK-92MI cells with an anti-CEA-specific single-chain variable antibody fragment (scFv). The anti-CEA-CAR-modified NK-92MI specifically recognized and efficiently eradicated CEA-expressing tumour cells. Furthermore, by pharmacologically-inducing CEA-upregulation, the cytotoxicity of these modified-NK cells was enhanced. These observations show the promise for anti-CEA-CAR-modified NK-92MI cells in clinical therapy for terminal-stage colorectal cancer treatment.

## Methods

### Cell and culture media

NK-92MI cells were incubated in an alpha modification of Eagle’s Minimum Essential Medium (α-MEM) from Gibco Laboratories (Gaithersburg, MD, USA) supplemented with 1.5 g L^− 1^ sodium bicarbonate, 0.2 mM inositol, 0.02 mM folic acid, 0.01 mM 2-mercaptoethanol, 12.5% foetal bovine serum (FBS) (Invitrogen, Grand Island, NY, USA), and 12.5% horse serum (Sigma-Aldrich Corp., St. Louis, MO, USA). The human colorectal carcinoma cell lines used in this study were HCT116 (ATCC CCL-247), WiDr (ATCC CCL-218), and LS174T (ATCC CL-188). They were obtained from the Bioresource Collection and Research Center, Taiwan (BCRC). The HCT116 cells were cultured in McCoy’s 5A medium (Gibco Laboratories) containing 1.5 g L^− 1^ sodium bicarbonate, 4.5 g L^− 1^ glucose, 10 mM HEPES, 1.0 mM sodium pyruvate (90%), and 10% FBS (Invitrogen, Grand Island, NY). The WiDr- and LS174T cells were maintained in α-MEM (Gibco Laboratories) supplemented with 1.5 g L^− 1^ sodium bicarbonate and 10% FBS. K562 cells were grown in Iscove’s Modified Dulbecco’s Medium (Invitrogen, Grand Island, NY) containing 1.5 g L^− 1^ sodium bicarbonate and 10% FBS. All cells were grown in a humidified incubator at 37 °C under a 5% CO_2_ atmosphere.

### Generation of anti-CEA-CAR NK-92MI cells

It has been shown that mouse monoclonal antibody (mAb) T84.66 scFv binds to CEA with high specificity and affinity [33]. The coding domain sequences for the variable regions of the mAb T84.66 heavy (V_H_) and light (V_L_) chains [34] were amplified separately and assembled with a linker using an overlapping polymerase chain reaction (PCR). The cDNA encoding of CEA-specific scFv of mAb T84.66 was isolated by PCR using gene-specific primers. The V_L_ region was amplified by PCR using the primer T84.66-V_L_- (forward: 5’-GGGGCCCAGCCGGCCTCAGAGATGGAGACAGACACAC-3′; reverse: 5’-CGCCAGATCCGGGCTTGCCGGATCCAGAGGTGGAGCCTTTTATTTCCAGCTTGGTCC-3′) and the V_H_ region was amplified by using the primer T84.66-V_H_ (forward: 5’-CGGCAAGCCCGGATCTGGCGAGGGATCCACCAAGGGCGAGGTTCAGCTGCAGCAGT-3′; reverse: 5’-CCGCTCGAGCGGTGAGGAGACGGTGACTGAGGTTC). The construct was generated by cloning the sequences encoding anti-CEA scFv fragment and the hinge region of CD8α (amino acids 105–165) into the plasmid pcDNA3.1/V5-HIS©TOPO®TA vector (Invitrogen, Groningen, Netherlands). The CD3ζ chain (amino acids of the transmembrane and intracellular domains) was cloned into the plasmid GEM®-T vector (Promega, Madison, WI, USA). The complete CAR sequence was derived from the resulting pcDNA3.1-scFv (anti-CEA)-CD8α-CD3ζ construct and cloned into the SfiI- and ClaI restriction sites of a modified retroviral pLNCX vector kindly provided by S. R. Roffler, Institute of Biomedical Sciences (Academia Sinica, Taipei, Taiwan). The pLNCX contained a leader sequence and an HA tag. It was sequenced for identification and yielded pLNCX-scFv (anti-CEA antibody)-CD8α-CD3ζ. Both the recombinant retroviral vector pLNCX-scFv (anti-CEA antibody)-CD8α-CD3ζ and the pVSV-G plasmid (envelope plasmid) (Clontech Laboratories, Inc., Mountain View, CA, USA) were co-transfected into packaging cell line 293 (Clontech Laboratories) with lipofectamine 2000 (Invitrogen, Carlsbad, CA, USA). Retroviral supernatants were generated from the GP2–293 cell line in DMEM supplemented with 10% FBS. After a 48 h incubation at 37 °C, the supernatants were harvested and then filtered through a 0.45-μm low-protein-binding filter (Minisart NML; Sartorius Lab Instruments GmbH, Göttingen, Germany). They were then used to transduce NK-92MI cells for 24 h. The transduced NK-92MI cells were further screened by neomycin sulphate-G418 (500 μg mL^-l^).

### Cell treatments

HCT116 and WiDr human colorectal cancer cells were seeded in 6-cm tissue culture dishes at a density of 2.5 × 10^5^ cells/ml under normal culture condition for 24 h. The cells were subjected to increasing concentrations of either the HDAC-inhibitor, sodium butyrate (NaB) (0.1-1 mM) or the DNA methylation inhibitor 5-azacytidine (5-AZA) (1 μM) for 10 h and 72 h, respectively. A 1 μM 5-FU treatment for 24 h was established as positive control. Total CEA protein was detected by western blot and surface CEA-expression was detected by flow cytometry. Non-cytotoxic concentration-levels which induced higher CEA-expression levels were selected for NaB and 5-AZA, at 0.1 mM and 1 μM, respectively. These induced cultures were then used to determine the cytotoxicity of anti-CEA-CAR NK-92MI cells.

### Western blot

HCT116 and WiDr cells were treated with NaB (0.1 mM) for 10 h or 5-AZA (1 μM) for 72 h. These groups, along with an untreated control group, were lysed in a radioimmunoprecipitation assay (RIPA) buffer (Sigma-Aldrich Corp., St. Louis, MO, USA) containing EDTA-free Protease-Inhibitor Cocktail Tablets and Phosphatase-Inhibitor Cocktail Tablets (Roche Diagnostics, Monza, Italy). Total protein concentrations in the lysates was measured using a bicinchoninic acid protein concentration assay (Pierce Biotechnology, Rockford, IL, USA). Total protein (20 μg) was electrophoresed on 10% polyacrylamide gels, transferred onto Immobilon-P polyvinylidene fluoride membranes (EMD Millipore, Bedford, MA, USA), and blocked with Tris-buffered saline (TBS)-0.05% Tween 20 containing 5% non-fat milk at room temperature (25 °C) for 1 h. Filters were probed with anti-human CEA (Santa Cruz Biotechnology, Dallas, TX, USA) or anti-β-actin antibodies (Sigma-Aldrich Corp., St. Louis, MO, USA) at 4 °C in the same buffer overnight. After wash, the filter was probed with horseradish peroxidase-conjugated secondary antibodies (Jackson ImmunoResearch Laboratories, West Grove, PA, USA) at room temperature (25 °C) in the same buffer for 1 h. Blots were developed using a chemiluminescent detection system (ECL; GE Life Science, Buckinghamshire, UK). Proteins were visualised using enhanced chemiluminescence detection. This process was performed in triplicate, and quantitation of immunoblots was performed using Adobe Photoshop 7.0.

### Binding assay

CEA-binding activity was examined by western blotting. In brief, either anti-CEA-CAR NK-92MI or NK-92MI cells were incubated with recombinant human CEA protein (rCEA) (0.8 μg) for 4 h. The cells were washed in PBS then lysed with 1 mL ice-cold RIPA (Boston Bioproducts, Worcester, MA, USA) containing protease inhibitors (Roche Applied Science, Vilvoorde, Belgium). The ability of the chimeric anti-CEA receptors on the NK cells to bind with human rCEA was determined by immunoblotting. The membrane was hybridised with mouse anti-human CEA antibody supernatant (Santa Cruz Biotechnology) and horseradish peroxidase-conjugated secondary antibodies (Jackson ImmunoResearch Laboratories). Proteins were visualised using enhanced chemiluminescence detection. This process was performed in triplicate, and quantitation of immunoblots was performed using Adobe Photoshop 7.0.

### Phenotype analysis of cell surface CEA-CAR expression of NK-92MI cells

Flow cytometer was used to analyse the cell surface expression of human influenza hemagglutinin (HA)-tagged CEA-CAR in transduced anti-CEA-CAR NK-92MI cells. To evaluate the cell surface expression of HA-tagged CEA-CAR, transduced anti-CEA-CAR NK-92MI cells were labelled with anti-HA antibody (Abcam, Cambridge, UK) or IgG isotype antibody as control. For surface CEA staining, cancer cells were harvested and stained with mouse anti-human CEA-FITC (BD Biosciences, San Jose, CA, USA). Cells were analysed with a FACSCalibur flow cytometer (Becton Dickinson, Franklin Lakes, NJ, USA). The fluorescence intensities of at least 10^5^ cells were recorded and analysed using CellQuest Pro software (Becton Dickinson). Geometric mean was established as the mean fluorescence intensity (MFI).

### Phenotype analysis of NK cell surface marker expression of NK-92MI cells

The phenotypes of NK cell surface marker expression on transduced anti-CEA-CAR NK-92MI and parental NK-92MI cells were determined using a FACSCalibur flow cytometer (Becton Dickinson). The cells were stained with FITC-labelled NKG2D, CD45, and CD56 antibodies (BioLegend, San Diego, CA, USA). The fluorescence intensities of ≥10^5^ cells were recorded and analysed with CellQuest Pro software (Becton Dickinson). Geometric mean was established as the MFI.

### Cytotoxicity assay

The cytotoxic effects of anti-CEA-CAR NK-92MI cells were investigated with a CytoTox96® Non-Radioactive Cytotoxicity Assay (Promega, Madison, WI, USA) according to the manufacturer’s protocols. This technique is the colorimetric alternative to the standard 4-h ^51^Cr release assay. Briefly, target cells were co-cultured with anti-CEA-CAR NK-92MI cells at various effector/target ratios (E/T) including 10:1, 5:1, 1:1, or 0.5:1. They were planted into a round-bottom 96-well culture plate. Each well contained a final volume of 100 μL. The contents were mixed gently and centrifuged at 250×g for 5 min and then incubated at 37 °C under a 5% CO_2_ atmosphere for 24 h. Fifty microlitres of the supernatant in each well was then transferred to the corresponding well of a flat-bottom 96-well enzymatic assay plate. Fifty microlitres of CytoTox96® Reagent was added to each well and the plate was incubated at room temperature (25 °C) and protected from light for 30 min. Fifty microlitres of Stop Solution was then added to each well and the absorbances were measured at λ = 490 nm. The percentage of cytotoxicity for each E/T was calculated as (experimental culture medium background) - (effector cell spontaneous release - culture medium background) - (target spontaneous release - culture medium background) / (target maximum release - volume correction control - target spontaneous release - culture medium background) × 100.

### Cell proliferation assay

Cell viability was determined by a CellTiter96 aqueous one-solution cell proliferation assay according to the manufacturer’s instructions (Promega, Madison, WI, USA). In brief, 5 × 10^3^ cells were seeded into a flat-bottom 96-well enzymatic assay plate for 1 day before exposure to the various compounds. HCT116 and WiDr cells were treated with NaB (0.1 mM) for 10 h or 5-AZA (1 μM) for 72 h. The treated groups were used to determine the effect of CEA-overexpression in correlation with 5-FU resistance. Cells were simultaneously co-treated with either NaB (0.1 mM) or 5-AZA (1 μM) at various 5-FU concentrations (0 μM, 2.4 μM, 4.8 μM, 9.6 μM, 19.2 μM, and 38.4 μM) for 72 h. The IC_50_ values were defined as 50% cell growth inhibitory concentrations of the 5-FU treatment groups and MTS assay was performed to determine IC_50_. After 72 h, 20 μL CellTiter96 aqueous one-solution was added to each well. After 4 h, the UV-absorbance of each solution was measured at λ = 492 nm. This process was performed in triplicate.

### Animal study

Nine-week-old female SCID mice were subcutaneously injected with 2 × 10^6^ WiDr cells in their right-side dorsa. When the tumours reached a volume range of 100-200 mm^3^, the mice were segregated into five groups (control, NaB, NK-92MI, anti-CEA-CAR NK-92MI, and anti-CEA-CAR NK-92MI + NaB). NaB 200 mg/kg was injected intraperitoneally 5 days per week and 10^7^ anti-CEA-CAR NK-92MI cells were injected intraperitoneally twice per week for 2 cycles. Tumour sizes were measured weekly. Mice were sacrificed either after 15 days of treatment or when the tumour reached the maximum allowed volume of 1,000mm^3^. Tumours were stored at − 80 °C for western blot analysis.

### Statistical analysis

All experiments were performed at a minimum of 3 times and the data were expressed as means ± standard error of the mean (SEM). Statistical significance was determined using Student’s *t* test. All data was processed with Prism v. 5.0 (GraphPad Software, San Diego, CA, USA). A multiple linear regression analysis was used to compare the differences among the three groups after adjusting for the effects of cell generation, a potential confounding variable. To take into the repeated measurements’ dependence, multiple linear regression by GEE method was used to further compare the difference of tumour volumes between the various control groups (control, NaB, and NK-92MI) and the CAR-NK cell therapies group (anti-CEA-CAR NK-92MI and anti-CEA-CAR NK-92MI + NaB). Statistical significance was defined as *P* <  0.05.

## Results

### Expression of anti-CEA-CAR in NK-92MI cells

**Fig. 1:**
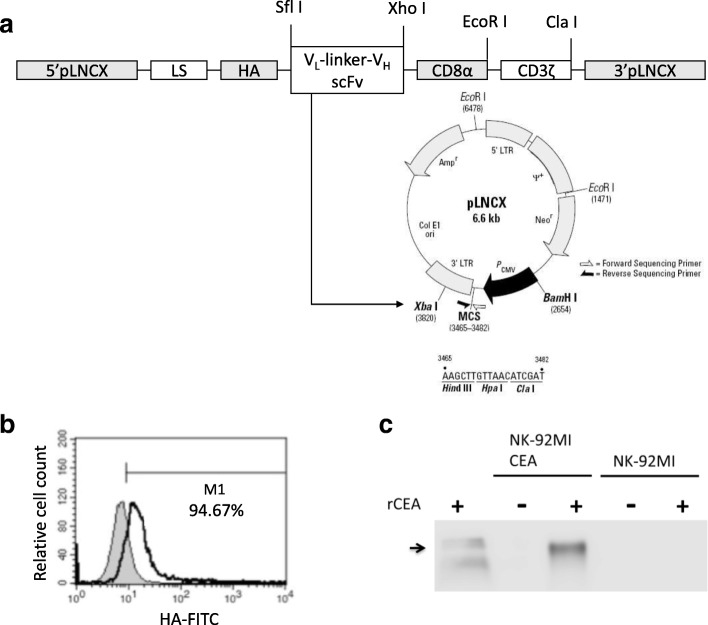
Genetic modification of NK-92MI cells with anti-CEA-CD8α-CD3ζ chimeric receptor. **a** Schematic image of the chimeric receptor anti-CEA-CD8α-CD3ζ. The chimeric receptor consisted of the V_L_ and V_H_ regions of the anti-CEA mAb joined to a CD8α and fused to the transmembrane and intracellular regions of human TCR-CD3ζ. Map of destination vector pLNCX wherein the cDNA for the fusion protein anti-CEA-CD8α-CD3ζ was cloned into SfiI and ClaI restriction enzyme sites of modified retroviral pLNCX vector containing leader sequence and HA tag and sequenced for identification. The product was pLNCX- anti-CEA scFv-CD8α-CD3ζ. Transfected cells expressing the transgene of interest were selected on cytocidal concentrations of neomycin sulphate (G418). **b** Surface expression of chimeric anti-CEA scFv-CD8α-CD3ζ. NK-92MI cells were analysed following staining with FITC-labelled HA tag Ab. Briefly, CAR expression was determined by flow cytometry with HA-tagged- and recognised anti-CEA chimeric receptor (green open area). Parental NK-92MI cells served as control (blue filled area). **c** Ability of anti-CEA chimeric receptor to recognise recombinant human CEA was determined by immunoblotting. Lysates of NK-92MI (lane 4) and transduced anti-CEA NK-92MI cells (lane 2) were separated by SDS-PAGE. Transduced anti-CEA NK-92MI or parental NK-92MI co-cultured with rCEA (lanes 3 and 5) were analysed by immunoblot analysis

To construct the anti-CEA specific CAR, the cDNAs of variable heavy-chain (V_H_) and light-chain (V_L_) domains of the humanised-monoclonal-anti-CEA antibody, the human influenza hemagglutinin (HA)-tag sequence, the CD8α hinge region, and the transmembrane and intracellular domains of CD3ζ were assembled stepwise into a pGEM-1 plasmid (Promega, Madison, WI, USA). The cDNAs were used to produce a scFv of the anti-CEA antibody. The complete CAR sequence was derived from the pcDNA3.1–1-anti-CEA scFv-CD8α-CD3ζ construct and cloned into pLNCX, a modified retroviral expression vector, to yield the pLNCX-based pL-anti-CEA scFv-CD8α-CD3ζ construct (Fig. 1a). NK-92MI cells were transduced with the anti-CEA scFv-CD8α-CD3ζ specific construct to generate anti-CEA-CAR NK-92MI cells and were repeatedly selected with G418 (500 μg mL^-l^). The cell surface expression of the anti-CEA-CAR in the transduced NK-92MI cells was investigated by staining with human influenza hemagglutinin (HA) tag-specific antibody recognising the HA-tag epitope incorporated into the extracellular domain of the chimeric receptor (Fig. 1b). The binding ability of the anti-CEA chimeric antigen receptor to recombinant human CEA protein was verified by western blotting. Transduced anti-CEA-CAR NK-92MI cells were cultured with 0.8 μg recombinant human CEA (rCEA) for 4 h. Lysate of the transduced NK-92MI cells cultured with rCEA was collected and analysed by immunoblotting (Fig. 1c, lane 3). Specificity was verified in parallel using a commercially available rCEA (Fig. 1c, lane 1).

### Phenotype of the anti-CEA-CAR NK-92MI cells

**Fig. 2:**
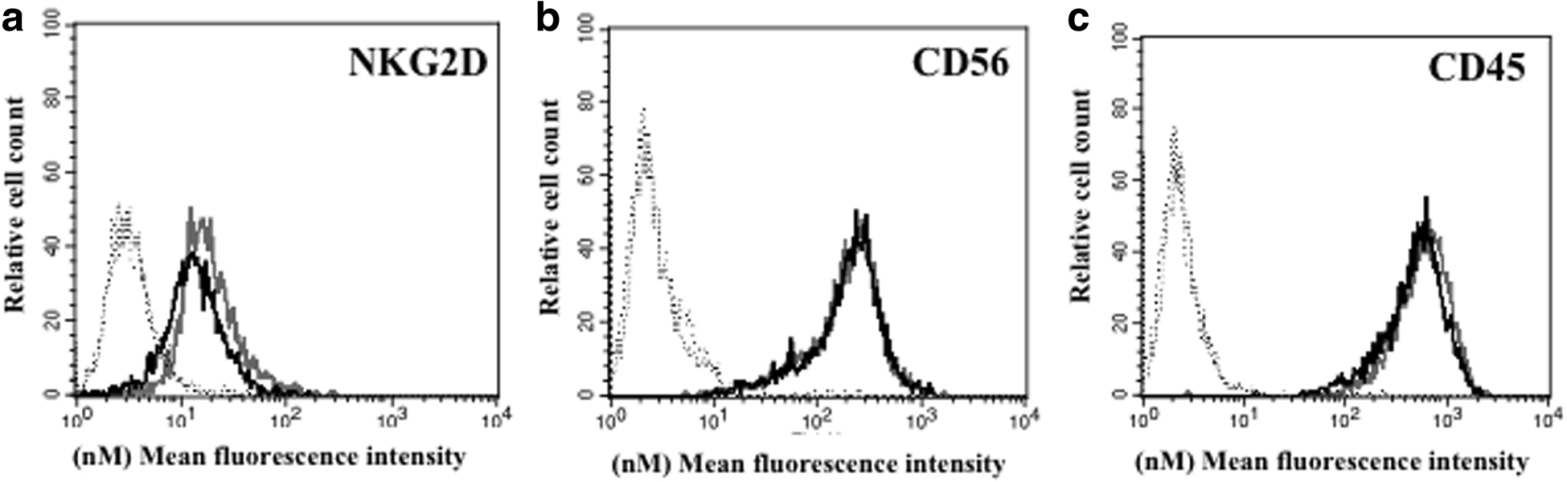
Phenotypic characterization of genetically-modified NK-92MI cells. Surface expression levels of various NK-92MI activation receptors were measured by flow cytometry. There was no significant difference between anti-CEA-CAR NK-92MI and parental NK-92MI cells in terms of (**a**) NKG2D, (**b**) CD56, and (**c**) CD45. Black line represents transfected anti-CEA-CAR NK-92MI cells. Grey line represents non-transfected NK-92MI cells. Dotted lines represent cells stained with isotype control

We investigated whether expression of the chimeric scFv receptor affected the NK-92MI phenotype. Flow cytometry was used to compare adhesion molecules (CD45 and CD56) and activation receptors (NKG2D) expressed by the anti-CEA-CAR NK-92MI cells with those of the parental NK-92MI. Separate experiments revealed no differences between the NK-92MI cells and the anti-CEA-CAR NK-92MI cells in terms of the expression levels of NK-92MI cell markers CD45, CD56, and NKG2D (Fig. 2). This data indicates that transduction of NK-92MI cells with scFv chimeric receptor did not phenotypically alter the expression levels of several important NK-92MI cell-associated markers.

### Detection of CEA-expression levels in various cancer cells lines

**Fig. 3:**
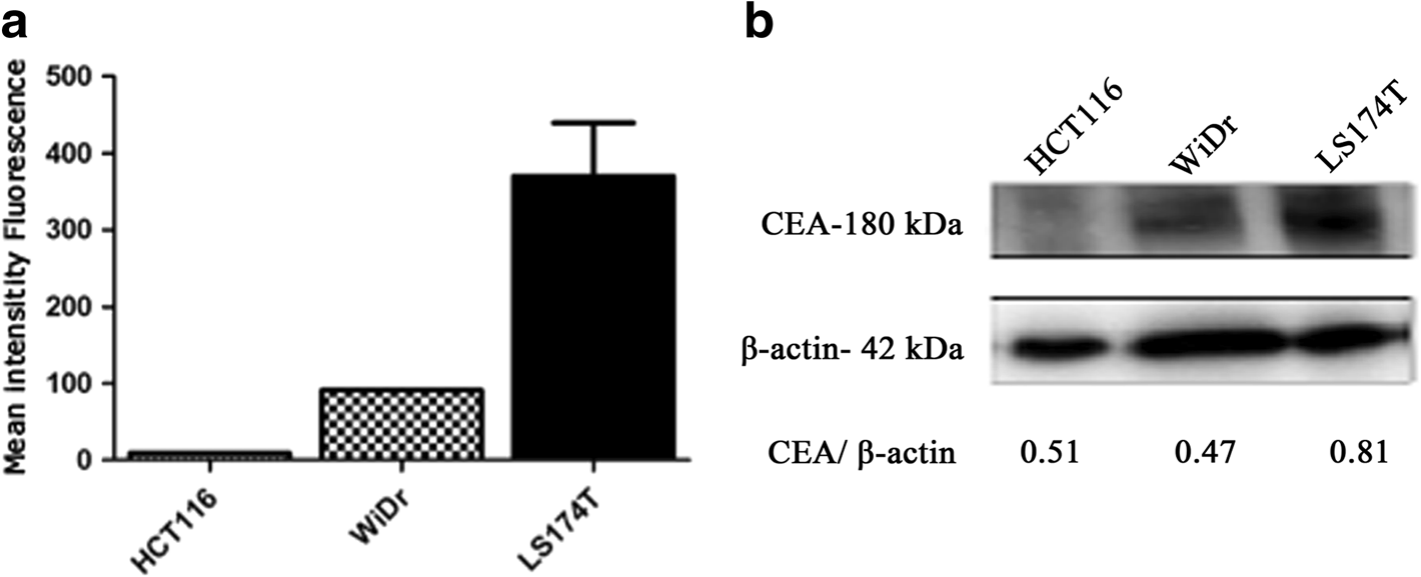
CEA-expression on colorectal carcinoma cell lines HCT116, WiDr, and LS174T. **a** Surface CEA-expression levels in colorectal carcinoma cell lines HCT116, WiDr, and LS174T were monitored by flow cytometry. **b** Determination of CEA protein expression by immunoblotting analysis in colorectal carcinoma cell lines

To assess the surface CEA-expression on various human colorectal cancer cell lines (HCT116, WiDr, and LS174T), intact cells were stained with a human CEA-specific antibody followed by flow cytometry. LS174T was shown to have highest CEA-expression levels, whereas expression levels were moderate in WiDr, and low in HCT116 (Fig. 3a). Relative differences in total CEA protein levels were confirmed by immunoblotting analysis. Human CEA-expressed protein was detectable in both WiDr and LS174T cells (Fig. 3b). Surface CEA-expression and CEA-secretion levels were found to have positive correlation (Fig. 3a). In contrast, CEA protein was almost undetectable in HCT116 cancer cells.

### Enhanced cytotoxicity of anti-CEA-CAR NK-92MI is correlated to surface CEA-expression on target cells

**Fig. 4:**
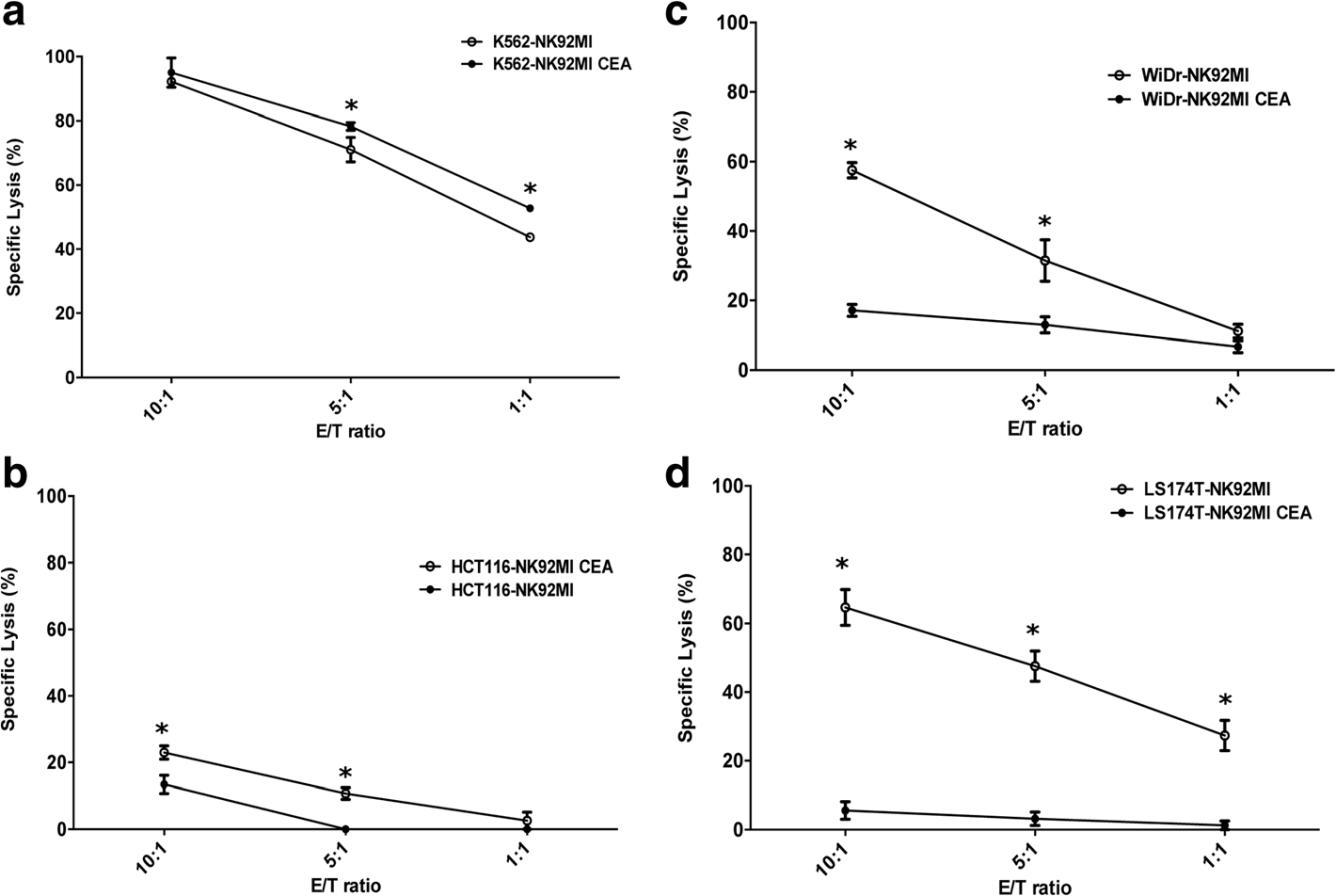
Anti-CEA-CAR NK-92MI recognise and kill CEA-expressing cells. Cytotoxicity of parental NK-92MI (●) or anti-CEA-CAR NK-92MI (○) against (**a**) human K562 erythroleukemia cells, (**b**) low-CEA-expressing HCT116 cells, (**c**) mildly CEA-expressing WiDr cells, and (**d**) highly CEA-expressing LS174T cells. Cells were incubated either with parental or anti-CEA-CAR NK-92MI at various effector/target (E/T) ratios for 4 h. Tumour lysis was determined from the release of LDH and measured by CytoTox96® non-radioactive cytotoxicity assay. Representative data from experiments conducted independently in triplicate are shown. *P <  0.05

NK-92 cell lines are highly cytotoxic against various malignant cells such as those found in leukaemia, lymphoma, and myeloma [35]. To determine whether genetic manipulation altered intrinsic NK cytotoxicity, the cell-killing activities of anti-CEA scFv-CD8α-CD3ζ NK-92MI and parental NK-92MI cells against different tumour cell lines were compared. The cytotoxicity of the transduced anti-CEA-CAR NK-92MI cells against the NK cell-sensitive target cell line K562 did not significantly differ from that of parental NK-92MI (Fig. 4a). This result shows that the process of transduction and gene modification does not diminish the native-cytotoxicity of parental NK-92MI cells. Anti-CEA-CAR NK-92MI cells failed to lyse low CEA-expressing HCT116 cells even at a high E/T ratio (specific lysis 23.71 ± 5.23% at E/*T* = 10:1) (Fig. 4b). Moderate CEA-expressing WiDr cells were found to sustain high cytotoxicity (specific lysis 57.51 ± 4.95% at E/T = 10:1; 31.14 ± 16.92% at E/*T* = 5:1). High CEA-expressing LS174T cells sustained even greater cytotoxicity (specific lysis 64.68 ± 9.01% at E/T = 10:1; 47.54 ± 12.60% at E/T = 5:1) (Fig. 4c and d). Even with E/T ratio decreased to 1:1, anti-CEA-CAR NK-92MI cells specifically and efficiently lysed LS174T cells (specific lysis 27.34 ± 7.68% at E/T 1:1), evidently due to high CEA-expression (Fig. 4d). Specific lysis at these conditions, however, was significantly reduced for moderate CEA-expressing WiDr cells (specific lysis 11.13 ± 1.378% at E/T = 1:1) (Fig. 4c). In contrast, all test groups were slightly sensitive, or insensitive altogether, to parental NK-92MI. These results suggest that after gene-modification, NK-92MI gain the capability to recognize and kill CEA-expressing cancer cells in a CEA-dependent manner.

### NaB and 5-AZA induced CEA-expression in human colorectal cancer cells

**Fig. 5:**
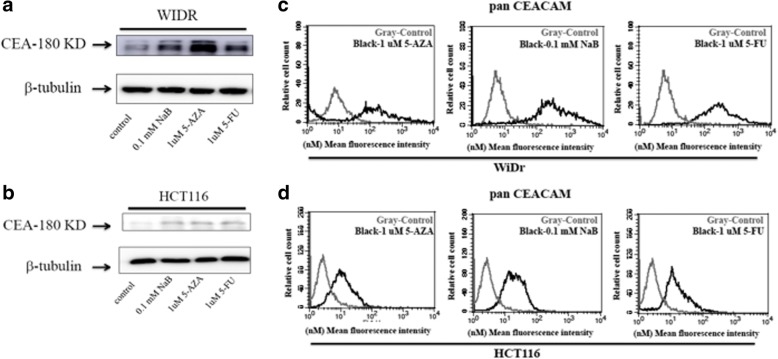
Quantification of surface and total CEA-expression in HCT116 and WiDr colorectal cancer cells after NaB and 5-AZA treatments. Low-CEA-expressing HCT116 cells and moderate-CEA-expressing colorectal cancer cells were treated with NaB (0.1 mM) for 10 h and 5-AZA (1 μM) for 72 h. Treatment with 5-FU (1 μM) for 24 h was used as positive control. Treatments with either NaB or 5-AZA induced the expressions of total CEA protein (**a** and **b**) and surface CEA (**c** and **d**) in both HCT116 and WiDr cells

To further confirm whether CEA-specific anti-CEA-CAR NK-92MI cell cytotoxicity was CEA-dependent, we pharmacologically-induced CEA-expression in either low-CEA-expressing HCT116 cells or moderate-CEA-expressing WiDr cells. HCT116 and WiDr cell lines were independently treated with NaB (0.1 mM) for 10 h or 5-AZA (1 μM) for 72 h. Both HCT116 groups treated either with NaB or separately treated with 5-AZA showed significantly increased CEA-secretion (Fig. 5b), and surface CEA-expression (Fig. 5d). Both WiDr groups treated either with NaB or separately treated with 5-AZA showed increased CEA-secretion (Fig. 5a), and surface CEA-expression (Fig. 5c).

### CEA-expression induced by NaB and 5-AZA enhanced cytotoxicity mediated by anti-CEA-CAR NK-92MI cells

**Fig. 6:**
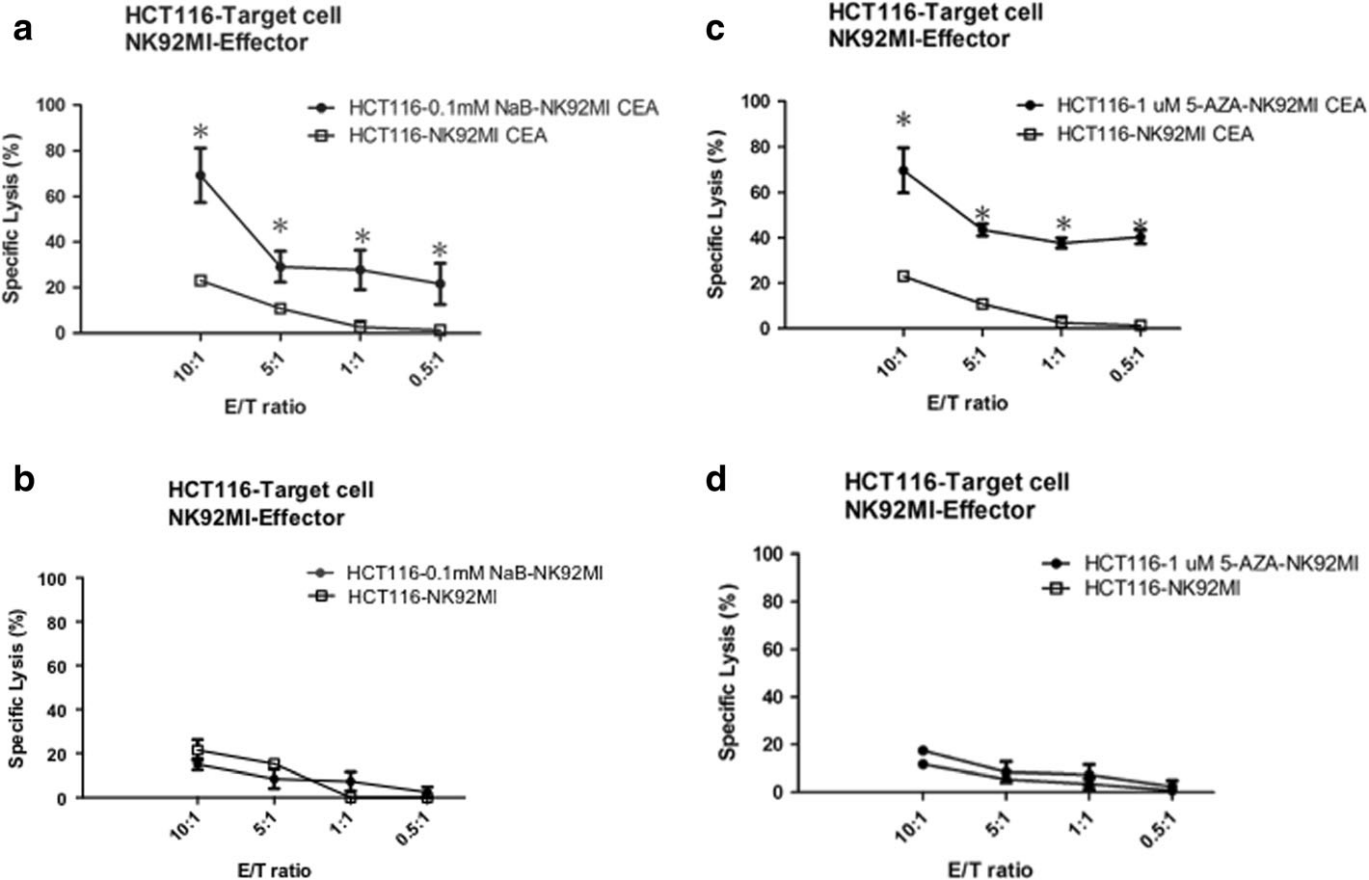
Increased specific lysis of HCT116 colorectal cancer cells by anti-CEA-CAR NK-92MI after treatments with NaB and 5-AZA. Retrovirally transduced anti-CEA-CAR NK-92MI were co-cultivated with HCT116 cells. Either NaB (0.1 mM) (**a**) or 5-AZA (1 μM) (**c**) treatment significantly enhanced the antigen-specific killing power of anti-CEA-CAR NK-92MI in HCT116 cells. HCT116 cells were also co-cultured with parental NK-92MI cells after treatment with either NaB (0.1 mM) for 10 h (**b**) or (1 μM) for 72 h (**d**). Representative data from experiments conducted independently in triplicate are shown. *P <  0.05

**Fig. 7:**
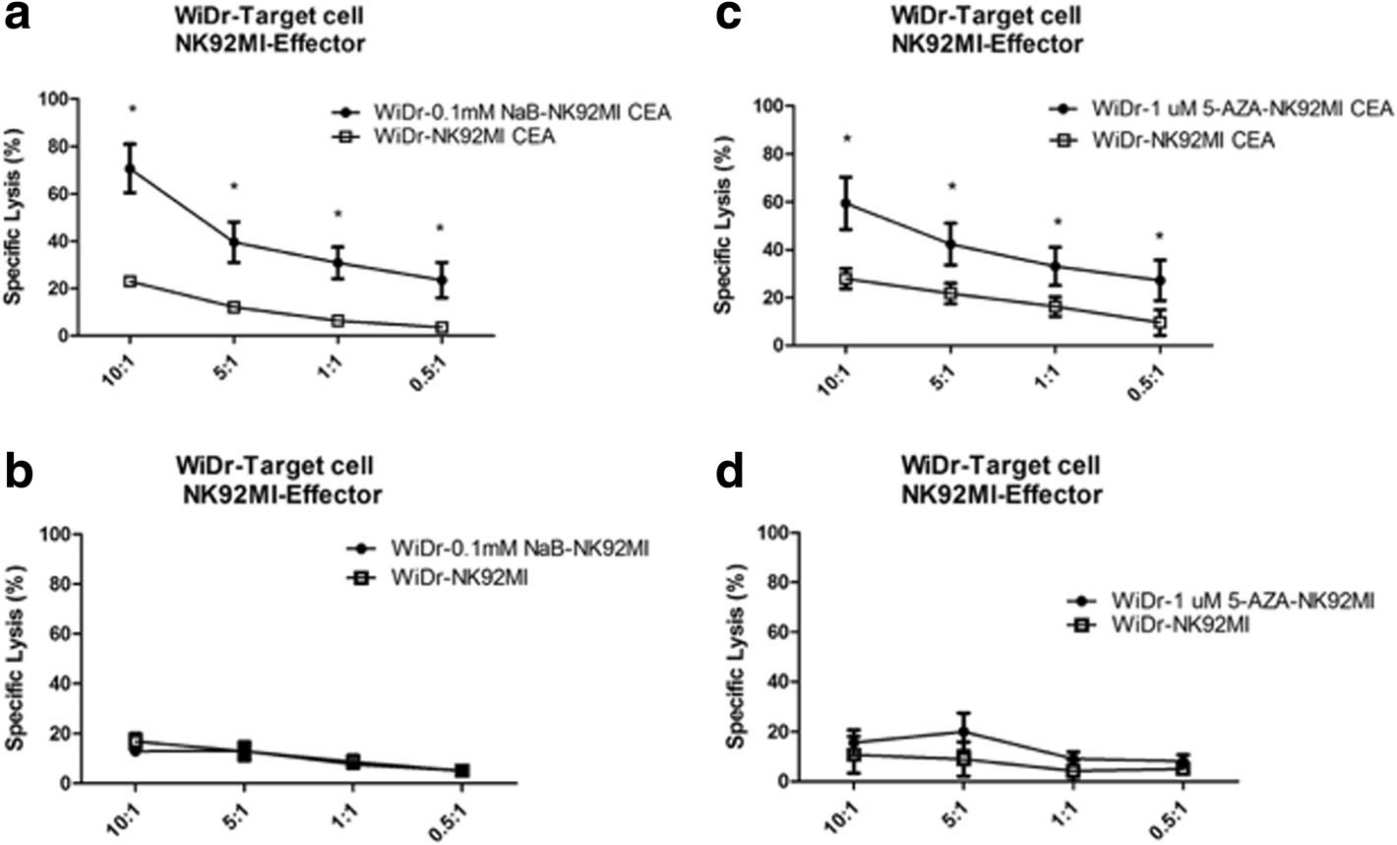
Increased specific lysis of WiDr colorectal cancer cells by anti-CEA CAR NK-92MI after NaB and 5-AZA treatments. Retrovirally transduced anti-CEA CAR NK-92MI cells were co-cultivated with WiDr cells. Either NaB (0.1 mM) (**a**) or 5-AZA (1 μM) (**c**) treatment significantly enhanced the antigen-specific killing power of anti-CEA CAR NK-92MI in WiDr cells. WiDr cells were also co-cultured with parental NK-92MI cells after treatment with either NaB (0.1 mM) for 10 h (**b**) or 5-AZA (1 μM) for 72 h (**d**). Representative data from experiments conducted independently in triplicate are shown. *P <  0.05

Previously, experiments had shown that anti-CEA-CAR NK-92MI cytotoxicity was CEA-dependent. Follow-up experiments then showed that nontoxic doses of NaB and 5-AZA induced total CEA-expression in both HCT116 and WiDr colorectal cancer cells (Fig. 5). We hoped to further investigate the effects of anti-CEA-CAR NK-92MI cytotoxicity on pharmacologically-induced CEA-expression colorectal cancer cells. HCT116 cell line, with its inherent low CEA-expression, was found that have significantly increased CEA-expression after treatment with NaB (0.1 mM) for 10 h or 5-AZA (1μM) for 72 h. These groups were then co-cultured with anti-CEA-CAR NK-92MI cells. Relative to the parental HCT116 treated by anti-CEA-CAR NK-92MI (specific lysis 22.99 ± 2.04% at E/*T* = 10:1; 10.71 ± 1.75% at E/*T* = 5:1) as control, we found pharmacologically-induced groups to have significantly-increased cell death levels. NaB-induced HCT116 groups had specific lysis of 69.20 ± 11.92% at E/T = 10:1 and 29.08 ± 6.81% at E/T = 5:1. The 5-AZA-induced groups had specific lysis of 69.70 ± 9.93% at E/T = 10:1 and 43.52 ± 2.67% at E/T = 5:1 (Fig. 6a and b). The WiDr cell line showed similar effects. NaB-induced WiDr group had specific lysis of 70.69 ± 10.19% at E/T = 10:1 and 39.56 ± 8.54% at E/T = 5:1. The 5-AZA-induced groups had specific lysis of 59.44 ± 10.92% at E/T = 10:1 and 42.37 ± 8.73% at E/T = 5:1 (Fig. 7a and b). On the other hand, there were no significant differences between the drug-treated groups (NaB 0.1 mM and 5-AZA 1μM) and the non-pharmacologically-treated control groups in terms of the cytotoxicity of parental NK-92MI cells against colorectal cancer cells (Figs. 6b, d, 7b, d). Whereas pharmacologically-enhanced CEA-expression had limited effect on parental NK-92MI cytotoxicity, NaB and 5-AZA sensitized and/or enhanced cytotoxicity of anti-CEA-CAR NK-92MI. This further confirms our previous findings that cancer cell killing effect of anti-CEA-CAR NK-92MI is positively-correlated to CEA-expression.

### Increased CEA-expression induced by NaB and 5-AZA correlated with 5-FU resistance

It has been reported CEA-expression levels are positively correlated to colorectal cancer cell resistance to 5-FU chemotherapy [31]. The effect of pharmacologically-enhanced CEA overexpression on 5-FU resistance was investigated.

HCT116 and WiDr cells were treated with NaB (0.1 mM) or 5-AZA (1μM) plus various 5-FU concentrations (1.2 μM, 2.4 μM, 4.8 μM, 9.6 μM, and 19.2 μM) for 72 h. We treated HCT116 cells independently with 5-FU, and found IC_50_ 4.39 ± 3.10 μM. We then applied 5-FU with NaB or 5-FU with 5-AZA, and found increased IC_50_, to 9.40 ± 6.03 μM and 11.76 ± 9.05 μM, respectively (Table 1). Levels measured by MTS Assay.

**Table 1:** NaB and 5-AZA-induced resistance to anti-cancer drug-5-FU in HCT116 and WiDr cells

Cell line	Treatment	IC50 of 5-FU (uM) Mean ± SD*	*P*-value**
HCT116	5-FU	4.39 ± 3.10	
	0.1 mM NaB+5-FU	9.40 ± 6.03	*<0.05(0.036)*
	1 μM 5-AZA+5-FU	11.76 ± 9.05	*<0.05(0.020)*
WiDr	5-FU	4.67 ± 0.55	
	0.1 mM NaB+5-FU	9.20 ± 2.74	*<0.001*
	1 μM 5-AZA+5-FU	10.81 ± 3.34	*<0.001*

*Results are presented as mean± SD of three independent experiments, each done in triplicate.

**The groups treated with 5-FU and NaB or 5-FU and 5-AZA were significantly increase IC50 comparing to 5-FU alone treatment group in HCT116 and WiDr cells

WiDr cells showed similar pattern, IC_50_ 4.67 ± 0.55 μM when treated with 5-FU. The groups treated with 5-FU and NaB or 5-FU and 5-AZA, IC_50_ was 9.20 ± 2.74 μM and 10.81 ± 3.34 μM, respectively (Table 1). Levels measured by MTS Assay.

### In vivo evaluation of the therapeutic efficacy of combination of anti-CEA-CAR NK-92MI cells and NaB

**Fig. 8:**
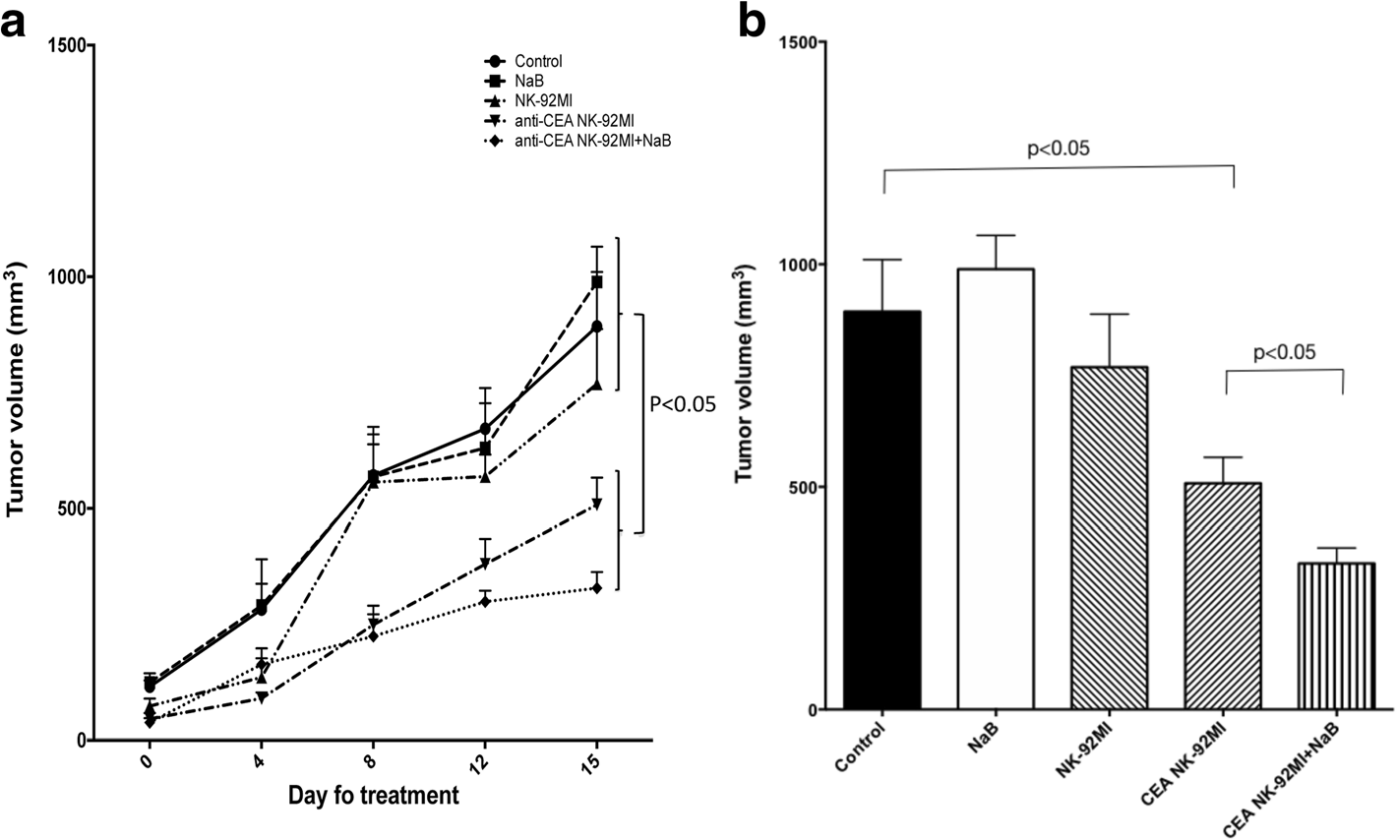
WiDr-injected SCID mice were treated with NaB, NK-92MI, anti-CEA CAR NK-92MI, and anti-CEA CAR NK-92MI + NaB for 15 days. Tumours were measured at Days 0, 4, 9, 12 and 15 after treatment. **a** Tumour growth curves show that the anti-CEA CAR NK-92MI and anti-CEA CAR NK-92MI + NaB treatments inhibit tumour growth significantly better than the control, NaB alone, or NK-92MI alone. **b** Treatment with NaB or NK-92MI did not significantly inhibit tumour growth relative to the control. Tumour size was significantly reduced by treatment with anti-CEA CAR NK-92MI and even more so in response to the combination of anti-CEA CAR NK-92MI and NaB

The therapeutic effect of anti-CEA-CAR NK-92MI cells was further confirmed by in vivo xenogeneic mice model. Tumour growth curves and sizes by Day 15 are shown (Fig. 8). There was no therapeutic effect in groups treated with NK-92MI or with NaB alone. However, anti-CEA-CAR NK-92MI cell therapy, with or without NaB, showed significant tumour growth-inhibition (*P* <  0.05) (Fig. 8a). The Day 15 tumours treated with anti-CEA-CAR NK-92MI cells were significantly smaller (508.19 ± 58.64 mm^3^) than the control groups (untreated, with NaB-alone, or with NK-92MI-alone) (893.7 ± 116.7 mm^3^) (*P* <  0.05). Combination treatment of anti-CEA-CAR NK-92MI cells with NaB showed even smaller tumour volumes (328.7 ± 34.92 mm^3^), with relative significance to control (*P* <  0.05).

In Table 2, multiple linear regression by GEE method showed that tumour volume in the control groups, had significant tumour volume increases, with averages of 142.59, 457.89, 523.35, 792.51, and 1138.87(mm^3^) at Days 4, 8, 12, 15, and 19, respectively. In the anti-CEA-CAR NK-92MI cell therapy groups, tumour volume growth was significantly smaller, with average decrease to controls of 57.67, 262.75, 225.25, 415.92, and 582.99(mm^3^) less than those of the various control groups at Days 4, 8, 12, 15, and 19, respectively. The differences reached significance at Day 8. All *p*-values< 0.001.

**Table 2:** Comparing the differences of the tumor volumes between the various control groups and the CEA CAR-NK cell therapies group

Parameter	B	Std. Error	95% Wald Confidence Interval		Hypothesis Test		
			Lower	Upper	Wald Chi-Square	df	Sig.
(Intercept)	109.135	10.4772	88.600	129.670	108.502	1	< 0.001
Group 1 vs. 0	−67.294	12.6052	−91.999	−42.588	28.500	1	< 0.001
Day 19 vs. 0	1138.866	120.2273	903.225	1374.508	89.730	1	< 0.001
Day 15 vs. 0	792.512	54.7220	685.259	899.766	209.743	1	< 0.001
Day 12 vs. 0	523.350	43.5205	438.052	608.649	144.609	1	< 0.001
Day 8 vs. 0	457.892	37.7332	383.936	531.848	147.258	1	< 0.001
Day 4 vs. 0	142.592	37.5555	68.984	216.199	14.416	1	< 0.001
Group x Day 19	−582.990	147.3587	− 871.808	−294.172	15.652	1	< 0.001
Group x Day 15	−415.924	68.5264	− 550.233	−281.615	36.839	1	< 0.001
Group x Day 12	−225.254	51.5495	−326.289	−124.219	19.094	1	< 0.001
Group x Day 8	−262.753	47.9948	− 356.821	− 168.685	29.971	1	< 0.001
Group x Day 4	−57.667	44.7379	−145.352	30.017	1.662	1	0.197

Group = 0 Various Controls Group; =1 CEA CAR-NK Cell Therapies Group

## Discussion

In this study, we determined that anti-CEA-CAR NK-92MI cells target CEA-positive tumour cells in a CEA-expression-dependent manner. Epigenetic modifiers which increased CEA-expression further enhanced cytotoxicity. High-expression CEA cells are generally resistant to 5-FU chemotherapy. To our knowledge, this is first demonstration of a collateral sensitivity strategy to salvage 5-FU-resistant CEA-expressing cells by utilization of CEA-targeting immunotherapy.

CAR-T-cell therapy has emerged as a potentially effective approach for the treatment of metastatic colorectal cancer [36, 37]. However, CAR-T treatments are associated with adverse events [18] and off-target effects [38], mostly commonly eliciting Cytokine Release Syndrome (CRS), a systemic inflammatory response that can lead to widespread organ dysfunction and death. The off-target effects generally associated with CAR-T were not observed in our study with NK or NK-92 cells, which lead us to believe NK and NK-92MI, specifically, to have less off-target concerns. References also show when compared to CAR-T, “CAR-NK-92 show ‘on-tumour’ in the absence of ‘off-target’ effects,” and “No concern about persisting CAR associated side effects” [15, 39]. Moreover, in clinical trials, patients with metastatic colorectal cancer receiving adoptively transferred autologous CEA-specific CAR-T experienced dose-limiting toxicity and severe transient inflammatory colitis. This reaction could be attributed to the intense immune response of T-cells [37]. Therefore, NK cells may be alternative cytotoxic effectors of CAR-driven cytolysis for the following reasons [15]: (1) NK cells may be safer and more effective CAR effectors than T-cells. T-cells induce proinflammatory cytokines (including tumour necrosis factorα (TNFα), interleukin (IL)-1, IL-6, and others) which could trigger cytokine storms, (2) NK kill target cells using specific natural receptors or transduced CAR, (3) each NK cell can kill four target cells on average, and [40] (4) CAR-NK have a shorter lifespan and, therefore, possibly lower residing toxicity than CAR-T. In contrast to CAR-T, the short-lived NK lines have no need for a suicide gene system [21, 41].

There are several limitations of the adoptive transfer of primary NK cells. These include (1) autologous NK cells may be silenced when their inhibitory receptors interact with self-MHC antigens [42], and (2) NK cells constitute only 10% of all lymphocytes, so primary NK cell adoption is limited by hindrances to ex vivo expansion and variations among patients in terms of their NK cell activity [14]. To overcome these limitations, established NK cell lines are used instead of primary NK cells [14, 43]. Clonal NK cell lines may be an attractive option as effector cells for immunotherapy. It is relatively easier and more cost-effective to use them in clinical trials under GMP conditions [44]. The United States Food and Drug Administration (USFDA) has approved NK-92 for use in clinical trials. NK-92 has undergone extensive preclinical development and completed phase I trials in cancer patients. Several CAR-modified NK-92 cells have been developed and demonstrated strong cytotoxicity in preclinical models [9, 22–24, 42, 43, 45–47]. The NK-92MI cells used in the present study are highly cytotoxic to human melanoma cells, as are their parental NK-92 cells [12, 13]. We obtained a satisfactory transfection efficiency of NK-92MI to CAR NK-92MI.

CEA is expressed in colorectal as well several other cancer types. CEA is considered a valuable target according to immunotherapy literature [48–50]. In the present study, genetically-modified NK-92MI-scFv cells harboured the scFv sequence specific for CEA mAb T84.66 on their plasma membranes and, therefore, had a high affinity and specificity for CEA [49, 51, 52]. We also showed that our stably transfected anti-CEA-CAR NK-92MI retains its intrinsic characteristics of adhesion and an activation marker of NK-92MI cells. NK-92MI cells also retain their cytolytic activity against the K562 human erythroleukemic cell line. Transduced anti-CEA-CAR NK-92MI exerted significantly elevated cytotoxicity against CEA-positive colon cancer cell lines relative to parental NK-92MI cells. Consequently, the anti-tumour activity of anti-CEA-CAR NK-92MI was also significantly increased in high CEA-expressing LS174T colon cancer cells. In contrast, HCT116 cells expressing only low CEA levels remained insensitive. There is, then, obvious target selection, and it occurs in a CEA-dependent manner.

It has been reported that membrane CEA-expression is ≥2× higher than that of the released CEA [53]. Our in vivo and in vitro data (Fig. 4) demonstrated that the cytotoxicity of transduced anti-CEA-CAR NK-92MI was higher than that of parental NK-92MI. Therefore, surface CEA-expression is highly important. CEA-secretion levels may have only a very minor effect. The level of CEA-secretion may be associated with poor tumour responses to chemoradiotherapy and increased risks of relapse [31, 54]. Drug-resistant human colorectal adenocarcinoma tumours produce abnormally high levels of CEA per cell [55]. It has been reported that several anticancer drugs (cisplatin, aspirin, and 5-FU) induced CEA-expression. Upon treatment, drug-resistant LoVo colon cancer cells produced higher CEA levels than non-resistant cells [55]. Therefore, chemotherapy-induced CEA-expression levels may indicate more chemo-resistant status. We explored whether epigenetic modifiers like DNA methyltransferase-inhibitors and HDAC-inhibitors induced higher CEA-expression levels and, therefore increased CEA vulnerability to targeted immunotherapy. The results showed that NaB and 5-AZA induced CEA-expression in both WiDr and HCT116 colon cancer cells (Fig. 5). They also increased IC_50_ of 5-FU for both cell lines (Tables 1). Anti-CEA-CAR NK-92MI cells recognized low-CEA HCT116 colon cancer cells more efficiently after drug treatment in vitro. We also demonstrated tumour growth delay in a WiDr mouse model after treatment with anti-CEA-CAR NK-92MI plus NaB.

## Conclusion

We successfully transduced NK-92MI with a retroviral vector encoding an anti-CEA-specific chimeric receptor. The chimeric receptor expression, phenotype, and anti-CEA-CAR NK-92MI cell line cytotoxicity were all defined. The cells specifically recognised and lysed CEA-expressing cancer cells. In addition, the epigenetic-modifiers which increased CEA-expression in cancer cells may have also increased the CEA-targeted cytotoxicity of anti-CEA-CAR NK-92MI cells. CEA-density per cell is frequently induced after chemotherapy, thereby, a collateral sensitivity strategy may apply to clinical bedside, in which CEA-targeting NK cells salvage post-chemotherapy relapses.

## Abbreviations

5-AZA: 5-Azacytidine
5-FU: 5-Fluorouracil
CAR: Chimeric Antigen Receptor
CEA: Carcinoembryonic Antigen
CRC: Colorectal Cancer
DNAM-1: DNAX Accessory Molecule-1
FBS: Foetal Bovine Serum
HA: Human Influenza Hemagglutinin
HDAC: Histone Deacetylase
IFN-γ: Interferon-γ
IL: Interleukin
KIRs: Killer Cell Immunoglobulin-Like Receptors
MHC: Major Histocompatibility Complex
NK: Natural Killer
NKG2D: NK Group 2 Member D
PCR: Polymerase Chain Reaction
RIPA: Radioimmunoprecipitation Assay
scFv: Single-Chain Variable Fragment
TCR: T-Cell Receptor
α-MEM: Alpha Modification of Eagle’s Minimum Essential Medium
